# High-Performance Refractive Index and Temperature Sensing Based on Toroidal Dipole in All-Dielectric Metasurface

**DOI:** 10.3390/s24123943

**Published:** 2024-06-18

**Authors:** Jingjing Zhao, Xinye Fan, Wenjing Fang, Wenxing Xiao, Fangxin Sun, Chuanchuan Li, Xin Wei, Jifang Tao, Yanling Wang, Santosh Kumar

**Affiliations:** 1 School of Physics Science and Information Engineering, Liaocheng University, Liaocheng 252000, China; 2210110410@stu.lcu.edu.cn (J.Z.);; 2 Shandong Provincial Key Laboratory of Optical Communication Science and Technology, Liaocheng 252000, China; 3 Liaocheng Key Laboratory of Industrial-Internet Research and Application, Liaocheng 252000, China; 4 Institute of Semiconductors, Chinese Academy of Sciences, Beijing 100083, China; 5 College of Information Science and Engineering (ISE), Shandong University, Qingdao 266237, China; 6 Ningbo Xingke Metal Materials Co., Ltd., Ningbo 315000, China; 7 Department of Electronics and Communication Engineering, Koneru Lakshmaiah Education Foundation, Vaddeswaram, Guntur 522302, India

**Keywords:** toroidal dipole, optical sensor, all-dielectric metasurface, fano resonance

## Abstract

This article shows an all-dielectric metasurface consisting of “H”-shaped silicon disks with tilted splitting gaps, which can detect the temperature and refractive index (RI). By introducing asymmetry parameters that excite the quasi-BIC, there are three distinct Fano resonances with nearly 100% modulation depth, and the maximal quality factor (Q-factor) is over 10^4^. The predominant roles of different electromagnetic excitations in three distinct modes are demonstrated through near-field analysis and multipole decomposition. A numerical analysis of resonance response based on different refractive indices reveals a RI sensitivity of 262 nm/RIU and figure of merit (FOM) of 2183 RIU^−1^. This sensor can detect temperature fluctuations with a temperature sensitivity of 59.5 pm/k. The proposed metasurface provides a novel method to induce powerful TD resonances and offers possibilities for the design of high-performance sensors.

## 1. Introduction

Sensing, which can detect and sense a specific physical quantity or environmental parameter and acquire essential chemical and physical information, has emerged as a swiftly developing technology. Compared to electrical and chemical sensors, optical sensors exhibit significant promise for temperature monitoring [1], environmental monitoring [2], chemical monitoring [3,4], and food safety [5,6,7] due to resistance to electromagnetic interference, high sensitivity, and good electrochemical stability [8]. In recent years, metasurfaces, as artificially aligned arrays of periodic subwavelength structures, have been shown to have remarkable properties in manipulating electromagnetic waves, which can be used to modulate phase, amplitude, and polarization direction [9,10,11,12,13]. Meanwhile, the metasurfaces can measure the positional shift of the resonance wavelength due to changes in the surrounding effective refractive index. They have been widely used in high-performance sensors [14,15], optical switches [16], and optical modulators [17].

Moreover, metasurfaces can excite electric and magnetic Mie resonances such as a magnetic dipole (MD), electric dipole (ED), and toroidal dipole (TD) [18]. Based on the polarization current distribution, the toroidal dipole can be categorized into electrical TD and magnetic TD [19]. The electrical TD arises from the circular polarization current encircling the torus, represented by the electric dipole moment formed by linking the head and tail [20]. The magnetic TD arises from the polar current flowing along the meridian of the torus, represented by the magnetic dipole moment formed by linking the head and tail [21]. It is worth noting that the TD resonance intensity in plasma structures is suppressed due to the inherent Joule loss [22]. However, the high Q-factor TD response has been extensively applied in all-dielectric metasurfaces [23,24,25] due to its high laser damage threshold and low inherent resistive loss [26]. The proposed metasurface structure is widely used in nonlinear optics [27], lasers [28], and filters [29]. 

In optical applications, a strong connection exists between TD metasurfaces with high Q-factor and bound states in the continuum (BIC). BIC is a localized state supported by a continuum spectrum with zero linewidth [30,31]. With its electromagnetic near-field enhancement and exceptionally high Q-factor [15], BIC offers new ways to enhance matter and light interactions in the radiation continuum. When the spatial symmetry of the mode does not match the spatial symmetry of the external radiated wave, the mode cannot couple to the external radiation, leading to an infinite lifetime, which implies an infinitely high Q-factor. Introducing symmetry breaking into the structure allows for creating an external radiation channel, converting the symmetry-protected BIC into a quasi-BIC mode capable of radiating into the external continuum. This results in resonance linewidths and Q-factors being limited. Quasi-BIC leakage resonance has been successful in applications in optical emitting devices, optical filters, and the detection of biological and chemical nanomembrane analyzers [32,33]. It has been shown that there is a strong correlation between BIC and high Q-factor TD metasurfaces [34]. Specifically, the toroidal dipole bound state (TD-BIC) observed in the Fano resonance induced by the continuum exhibits characteristics of both TD and BIC.

This article introduces an all-dielectric metasurface consisting of silicon disks with tilted splitting gaps on a silica substrate. The metasurface exhibits three Fano resonance responses when illuminated by normally incident polarized light. Multipole decomposition and near-field analysis show that the double resonance excited by introducing an asymmetry in the near-infrared wavelengths originates from the TD resonance. The sensing performance of the structure is simulated at different refractive indices and temperatures, showing that the metasurface can serve as both a temperature sensor and a refractive index sensor, which is important in biomedical applications. In addition, the effects of incident light angle and geometrical parameters on the properties of the metasurface are investigated. The present work introduces a new method to excite strong TD quasi-BIC resonances.

## 2. Structural Design and Theoretical Analysis

**Figure 1:**
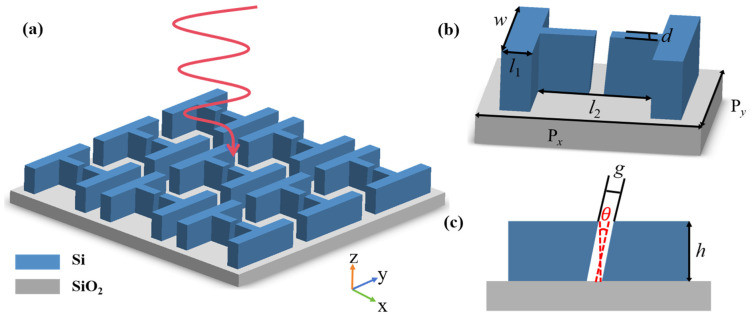
(**a**) Diagram of the metasurface array. (**b**) Diagram of the unit metasurface. (**c**) The *x*–*z* side view of the unit metasurface.

The proposed metasurface structure consisting of “H”-shaped silicon disks with tilted splitting gaps is shown in Figure 1. The initial geometric parameters are: the unit cycle of the metasurface is P*_x_* = P*_y_* = 750 nm, the height *h* of the silicon disc is 500 nm, and the tilt gap angle *θ* is 5°. The length *l_1_* of the nanorod is 105 nm, the length *l_2_* is 400 nm, the width *w* is 602 nm, the gap width *g* is 100 nm, and the gap length *d* is 80 nm. The FDTD solution is used to simulate and analyze the structural characteristics of the metasurface. Periodic boundary conditions are utilized in the *x* and *y* directions, while perfectly matched layers (PML) are applied in the *z* direction. The material parameters for Si and SiO_2_ can be found in the Palik refractive index database [35]. The structure is placed in the air with a refractive index of 1 and is illuminated by normally incident *x*-polarized light.

**Figure 2:**
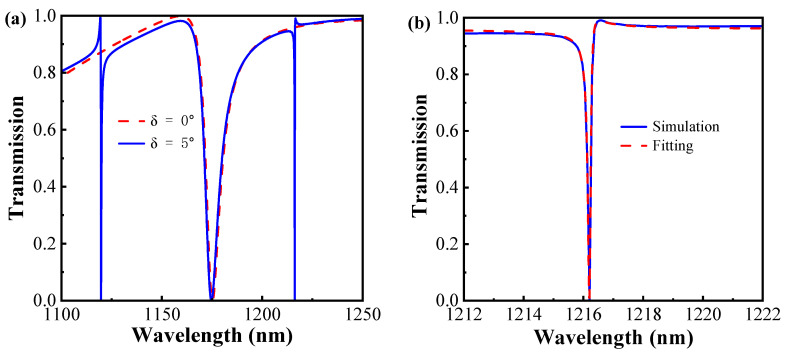
(**a**) The transmission spectra curves of metasurface structure at symmetry and symmetry breaking. (**b**) The blue solid line shows the simulation curve for the resonant mode FR3, and the red dashed line indicates the fitted curve.

The asymmetry factor is defined as the angle *θ* of the silicon splitting gap, which breaks the symmetry of the structure to excite the Fano resonance. Figure 2a illustrates the transmission spectra curves of the structure for both symmetry and symmetry breaking. The red curve shows a significant Fano resonance, called FR2, occurring at 1175.55 nm under the symmetric metasurface. When the asymmetry factor *θ* is 5°, the appearance of the double Fano resonance at 1119.71 nm and 1216.23 nm, as shown by the blue curves in Figure 2a, results from the creation of radiative channels between the free-space radiative and non-radiative bound states, named FR1 and FR3. Additionally, all curves achieve a spectral contrast ratio of approximately 100%, defined as:

(1) $$\frac{T_{peak}-T_{antipeak}}{T_{peak}+T_{antipeak}}\times 100\%$$

In Figure 2b, the transmission spectrum of the resonance mode FR3 is matched using the classical Fano equation [36,37]:

(2) $$T_{Fano}=\;|a_{1}+ja_{2}+\frac{b}{\omega -\omega_{0}+j\gamma }{|}^{2}$$

In Equation (2), the resonant frequency is represented by $\omega_{0}$, while $a_{1}$, $a_{2}$, and *b* are real numbers. Additionally,  γ  denotes the overall attenuation rate of the resonant cavity. For resonance mode FR3, $a_{1}\;$= 0.16714, $a_{2}\;$= 0.96508, *b* = 72.31972, *γ* = 0.0507 × 10^−3^ eV, and $\omega_{0}\;$ = 1.0217 eV. The method of calculating the Q-factor (Q) for the resonance peaks of the metasurface can be expressed as follows:

(3) $$Q=\frac{\omega_{0}}{2\gamma }$$

**Figure 3:**
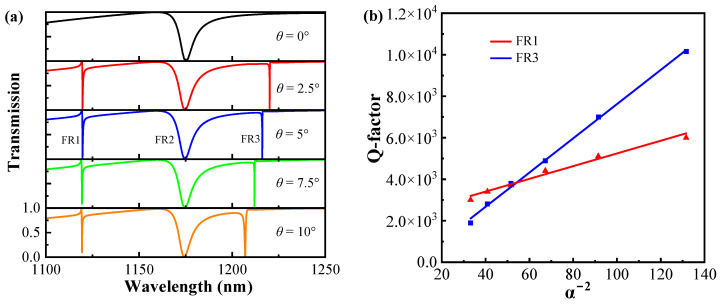
(**a**) Transmission spectra of metasurface at different values of tilt angle θ. (**b**) The correlation of the Q-factor and $\alpha^{-2}$ of the modes FR1 and FR3.

Figure 3a shows the variation of the transmission spectrum with the parameter *θ* from 0° to 10°. In a symmetric metasurface with *θ* equal to 0°, no Fano resonances occur at modes FR1 and FR3, and the resonant linewidth completely disappears, indicating no energy leakage from the bound state to the free-space continuum. Thus, the resonant modes FR1 and FR3 are supported by the symmetry-protected BIC state. As the tilt angle *θ* increases, new Fano resonances appear near 1119.71 nm and 1216.23 nm and exhibit an expansion of the line widths, which is due to the leakage of radiation channels between the metasurface and free space radiates the BIC energy, transforming the BIC mode into a high Q-factor and limited quasi-BIC mode [38,39]. Moreover, reducing the symmetry break narrows the radiation channel and decreases energy radiated into free space, thereby increasing the Q-factor [39,40]. In Figure 3b, the asymmetric parameter α (α = sin*θ*) is defined, to analyze the correlation between the $\alpha^{-2}$ and the Q-factor. The Q-factor gradually increases as α decreases, reaching infinity when α is 0, indicating a symmetric metasurface structure. The correlation between α and the Q-factor of Fano resonance modes FR1 and FR3 can be quantitatively expressed as follows [41]:

(4) $$Q\;{\propto \alpha }^{-2}$$

The result implies that the high Q-factor can be manipulated by θ. Altering θ essentially modifies the symmetry of the metasurface structure, thereby influencing the degree of magnitude of the Q-factor. 

**Figure 4:**
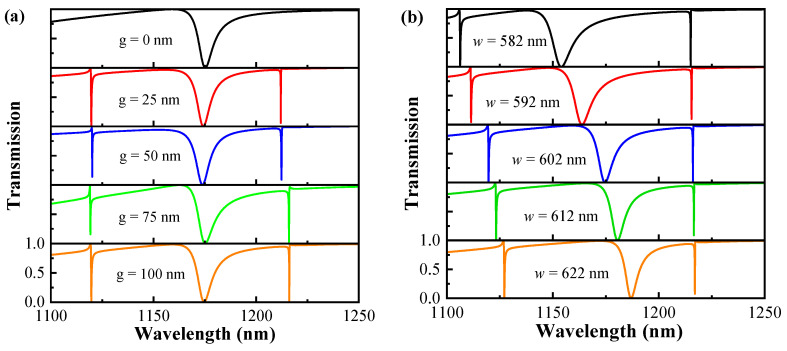
(**a**) The variation of the transmission spectrum with the splitting gap *g* when other parameters are held constant. (**b**) The variation of the transmission spectrum with the silicon disc width *w* when other parameters are held constant.

As previously mentioned, the resonance modes closely correlate with the asymmetric parameter *θ* of the metasurface. Hence, analyzing the influence of other structural parameters on the resonance is necessary. Figure 4 calculates the relationship between the transmission spectrum and the geometrical parameters of the structure, and an asymmetric structure with *θ* = 5° chosen for the analysis. Figure 4a depicts the variation of the transmission spectra for different splitting gaps *g*. The metasurface is symmetric at *g* = 0 nm. As *g* increases, resonance modes FR1 and FR3 with high Q-factors appear. The positions of modes FR1 and FR2 have not significantly shifted, but mode FR3 exhibits a slight red shift. Figure 4b shows the variation of the transmission spectra for silicon disc widths *w*, keeping other parameters constant. Both resonant modes FR1 and FR2 exhibit a significant red shift as *w* increases, and the resonant mode FR3 exhibits a slight red shift, which is due to the increase in the effective refractive index of the structure with increasing width *w* of the silicon disc. Thus, the parameter *w* does not affect the symmetry of the structure but influences the resonance wavelength shift of modes.

**Figure 5:**
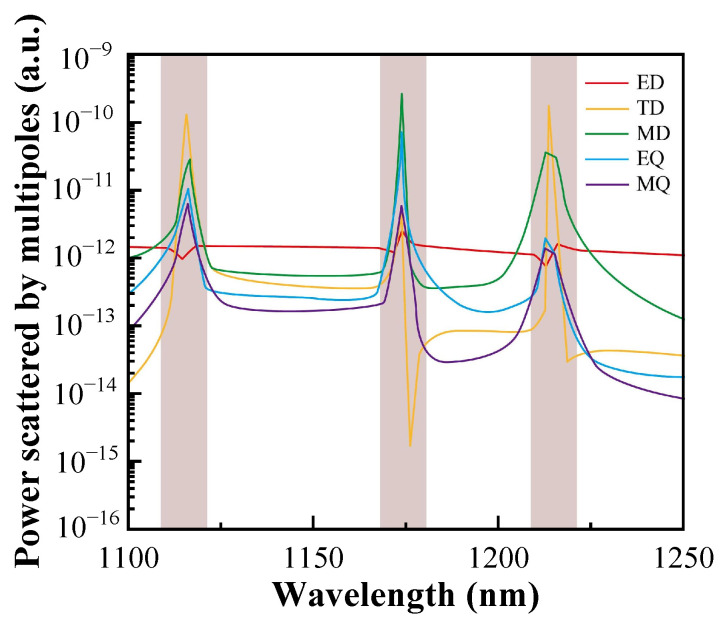
Multipole decomposition of three resonance modes.

To clearly understand the physical mechanism of the three resonances excited by this metasurface, the contributions of different multipoles to the resonance response, including the electric dipole (ED), the magnetic dipole (MD), the toroidal dipole (TD), the electric quadrupole (EQ), and the magnetic quadrupole (MQ), are evaluated in Figure 5 using the formulae in the Cartesian coordinate system [36,42,43].

(5) $$P=\frac{1}{i\omega }\int jd^{3}r$$

(6) $$M=\frac{1}{2c}\int (r\times j)d^{3}r$$

(7) $$T=\frac{1}{10c}\int [(r\times j)r-2r^{2}j]d^{3}r$$

(8) $$Q_{\alpha \beta }^{\left(e\right)}=\frac{1}{2i\omega }\int [(r_{\alpha }j_{\beta }+r_{\beta }j_{\alpha }-\frac{2}{3}(r\times j)\delta_{\alpha ,\beta }]d^{3}r$$

(9) $$Q_{\alpha \beta }^{\left(m\right)}=\frac{1}{3c}\int [{\left(r\times j\right)}_{\alpha }r_{\beta }+{\left(r\times j\right)}_{\beta }r_{\alpha }]d^{3}r$$

where α, β, and γ denote the directions of the *x*, *y*, and *z* axes, respectively, *c* is the speed of light in a vacuum, the spatial position vector is represented by r, ω denotes the angular frequency, and ***j*** is the current density. The multipole decomposition results reveal the primary multipole components near three resonance wavelengths, which are TD, MD and TD, respectively. The results show that the designed all-dielectric metasurface can excite quasi-BIC modes that support TD.

**Figure 6:**
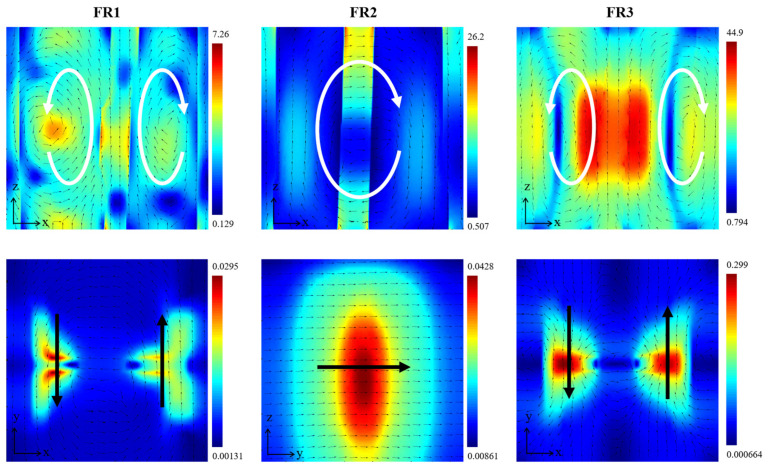
The near-field distributions of the normalized electric and magnetic fields of the nanostructure in the three resonance modes. The vectorial distribution of the electric field in the resonance modes is indicated with white arrows. The vector distribution of the magnetic field in the resonance modes is indicated with black arrows.

To further analyze the physical mechanisms of the resonant modes, the electromagnetic distributions of the three resonant modes are simulated, with white arrows indicating the electric field vectors and black arrows indicating the magnetic field vectors. As shown in Figure 6 for modes FR1 and FR3, the electric field in the *x*–*z* plane forms two opposing vortices, in the *x*–*y* plane forming a closed magnetic vortex magnetic field vectors circulating counterclockwise in the structure, indicating a typical toroidal dipole (TD) feature [21,44,45,46]. For mode FR2, the electric field forms a closed electric field vector circulating clockwise in the *x*–*z* plane, and the magnetic field vector is linearly aligned along the *y*-axis in the *y*–*z* plane, which can be recognized as a MD response along the *y* direction.

**Figure 7:**
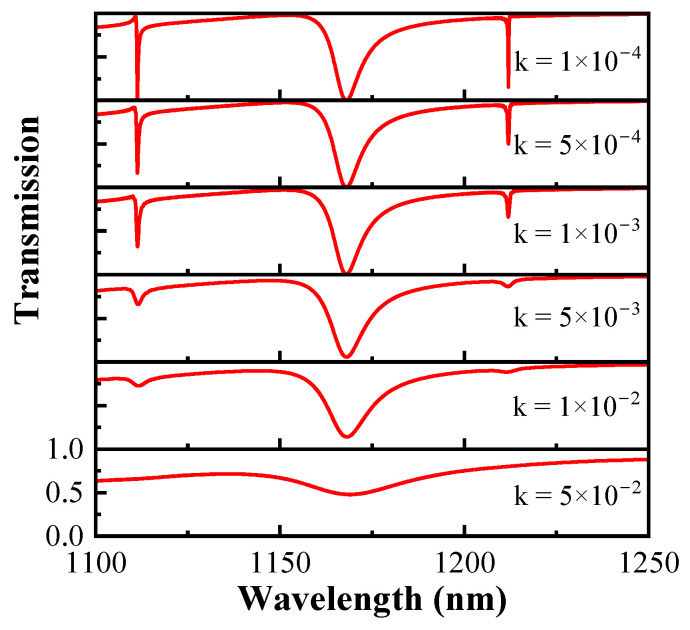
Transmission spectra of asymmetric structures at *θ* = 5° for different loss levels k.

With the proposed metasurface, the surface roughness of silicon will cause absorption and scattering losses, impacting the metasurface responses. Here, the imaginary part of the refractive index of silicon, i.e., the extinction coefficient k, is used to quantify the optical loss. Transmission spectra of asymmetric structures at *θ* = 5° for different loss levels k are shown in Figure 7. When k < 10^−4^, the metasurface resonance response is almost unaffected by the loss and maintains the original Fano resonance spectrum. With increasing k, the transmission waveform gradually deteriorates. FR1 and FR3 exhibit high sensitivity to optical loss, with the modulation depth and Q-factor experiencing significant reduction when k exceeds 5 × 10^−4^. However, FR2 is less affected. When k < 10^−2^, the Q-factor of the mode FR2 decreases, with no significant change in modulation depth. At k = 5 × 10^−2^, the standard Fano resonance spectral lines become difficult to observe due to the significant reduction in both modulation depth and the Q-factor of FR2. Indeed, the extent to which resonances are suppressed is similarly affected by the size of the asymmetric fracture. A metasurface with minor asymmetric breaks is more sensitive to the introduced losses [47].

## 3. Applications

**Figure 8:**
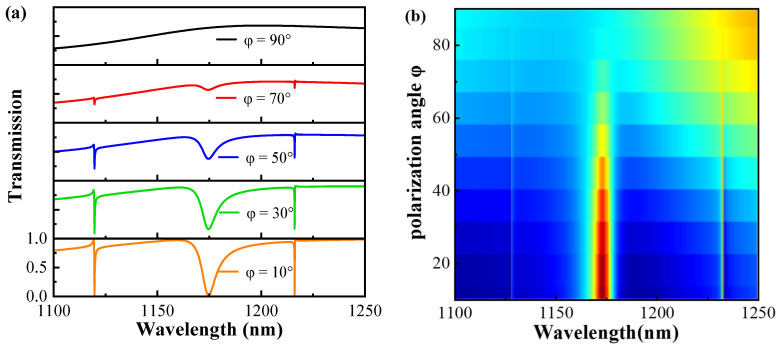
(**a**,**b**) Transmission spectra of asymmetric structures at different polarization angles φ.

The polarization dependence of the metasurface on the light source is investigated for potential applications in optical devices. Here, the polarization angle φ is the angle between the direction of polarization of the incident light and the *x*-axis. Figure 8 displays transmission spectra at various polarization angles φ for the asymmetric structure, which effectively modulates the transmission amplitude of the Fano resonance. The modulation depth of the three resonant modes decreases with increasing polarization angle without significant wavelength shift. At a polarization angle of 90°, the three resonant modes vanish. The analysis of the metasurface transmission spectra in Figure 8a,b shows that changing the polarization angle of the light source changes the modulation depth of the resonance peaks without affecting their wavelength positions. Therefore, the proposed metasurface depends on the incident polarization. By adjusting the polarization angle of the incident light, a switching state transition can be achieved, which is of great application in the field of optical switching.

**Figure 9:**
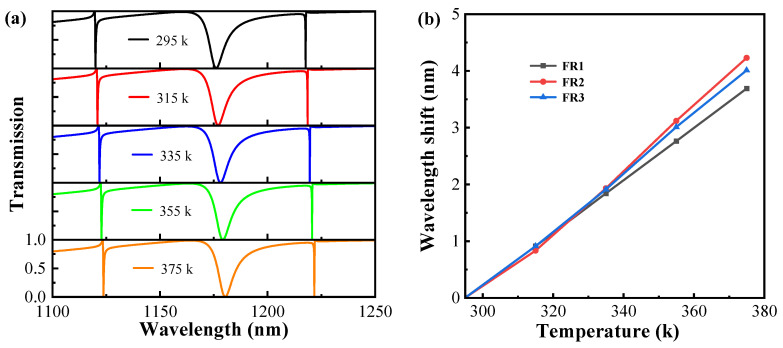
(**a**) Transmission spectra of resonant modes at various temperatures. (**b**) The relationship between wavelength shift and temperature is analyzed in three resonance modes.

The temperature-sensing properties of asymmetric metasurfaces are analyzed in Figure 9, where the refractive index of the material changes due to thermo-optic effects when the temperature in the environment where the structure is located changes. The correlation between the temperature and RI of the material can be defined as $n(T)=n(T_{0})+\gamma (T-T_{0})$, where the initial temperature $T_{0}$ is set to 295 K, and the thermal-optical coefficient is denoted by γ. The thermo-optic coefficients of Si and SiO_2_ obtained from experimental measurements are 2.01 × 10^−4^ and 8.40 × 10^−6^, respectively [48,49]. In Figure 9a, the transmission spectra vary as the temperature ranges from 295 K to 375 K. All resonance peaks are red-shifted. Figure 9b shows the wavelength shift with temperature for the three resonance modes. The temperature sensitivity can be defined as S(T) = ∆λ∕∆T, Δλ denotes the wavelength position shift, and ΔT denotes the temperature difference. The temperature sensitivities of the three resonance modes of the structure are calculated to be 46.5 pm/k, 59.5 pm/k, and 55 pm/k. 

**Figure 10:**
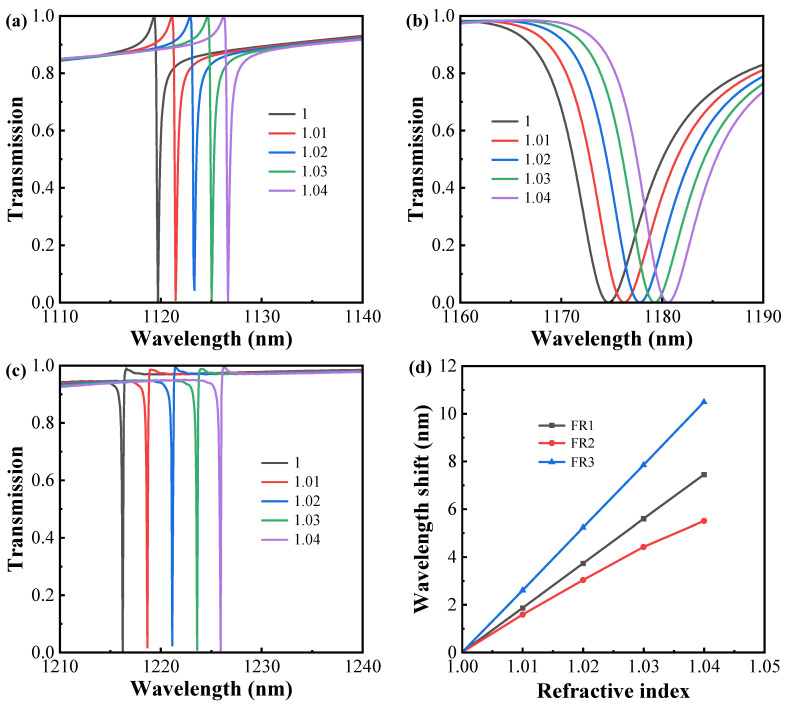
(**a**–**c**) Transmission spectra of the proposed metasurface structure with refractive indices of 1, 1.01, 1.02, 1.03, and 1.04, respectively. (**d**) Correlation between wavelength shift and refractive index.

In view of the high Q-factor and strong field enhancement effect of the proposed metasurface, it has excellent potential for a wide range of applications in the sensing field. Therefore, the performance of refractive index sensing is investigated by choosing an asymmetric structure with *θ* = 5°. As the refractive index of the surrounding environment ranges from 1 to 1.04, Figure 10a–c depicts three sharp Fano resonance modes in the transmission spectra. These resonance positions are significantly red-shifted. Figure 10d depicts the correlation between wavelength offset and refractive index, which is analyzed through simulation across three resonance modes. Two key performance indicators determine the excellence of a sensor. The RI sensitivity (S) of the sensor can be expressed as S =  ∆λ/Δn  (nm/RIU), Δλ  denotes the wavelength position shift, and Δn denotes the refractive index difference. The figure of merit (FOM) can be expressed as FOM = S/∂λ, representing the ratio of refractive index sensitivity (S) to resonant linewidth (∂λ). The evaluated S for the three Fano resonances is 186 nm/RIU, 158 nm/RIU, and 262 nm/RIU, with FOM values reaching 701 RIU^−1^, 19 RIU^−1^, and 2183 RIU^−1^, respectively. The difference in sensitivity between these resonant modes is mainly attributed to the field distribution. It has been demonstrated that adjusting the surrounding refractive index enables manipulation of the multipolar far-field scattering within dielectric particles, leading to a customized optical response [50,51]. It is worth noting that the above-evaluated parameters can be further enhanced by reducing the asymmetry if future fabrication techniques permit. Thus, the multi-Fano resonance proposed in this paper offers promising applications in multichannel biosensing. In addition, the simulated performance of this sensor is compared with other sensors, as shown in Table 1, indicating that the sensor has excellent sensing characteristics.

**Figure 11:**
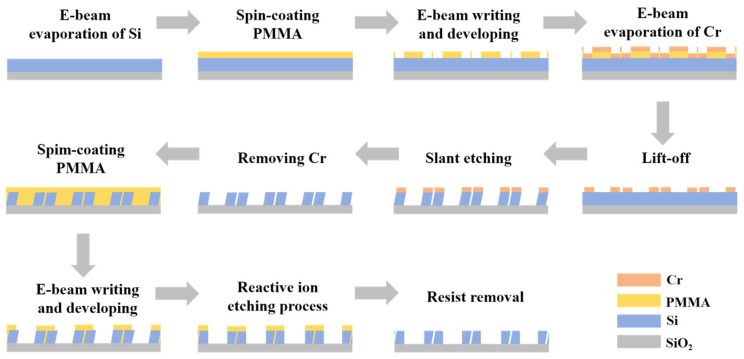
Manufacturing process for metasurface structures.

The feasibility of experimentally preparing an all-dielectric metasurface, which is compatible with popular nano-preparation techniques such as electron beam lithography and directional reactive ion beam etching, is analyzed. Figure 11 shows the fabrication scheme of the designed metasurface structure. First, silicon films are deposited on the substrate using electron beam evaporation, and a PMMA photoresist is spin-coated onto the silicon film. Next, the inverted nanostructures are obtained by inductively coupled plasma etching. Cr films are deposited by electron beam evaporation, and the patterns are transferred onto Cr tilt-etched Si films with Cr nanostructures as masks. The metasurface structure is achieved by removing the Cr mask with a Cr etchant. Then, the PMMA films are spin-coated onto the nanostructures. Subsequently, the structure is obtained by inductively coupled plasma etching using electron beam lithography, and the structure is transferred into the silicon layer by reactive ion etching. Finally, the photoresist is removed and cleaned with deionized water. The complexity of the designed structure itself makes its preparation challenging.

## 4. Conclusions

In summary, this article theoretically investigates the proposed all-dielectric metasurface consisting of tilted splitting gap silicon discs. The field distribution in the excited state Fano resonance mode at tilted gap angle *θ* = 5° exhibits two toroidal dipoles with high Q-factors, calculated by fitting to be 4225 and 10,135, respectively. The effects on the resonance modes are analyzed by adjusting the key geometrical parameters, in which the resonance modes are highly sensitive to the polarization angle of the light source, providing a broad application prospect for realizing optical switching. The maximum refractive index sensitivity of 262 nm/RIU and a figure of merit reaching 2183 for the sensor are obtained through simulation analysis using the optimized structural parameters. In addition, the thermo-optic coefficient of the material used in the metasurface enables the proposed sensor for temperature sensing with a sensitivity of 59.5 pm/k. It is noteworthy that, by adjusting the geometrical parameters of the structure, the resonant wavelengths of the two modes can be optimized for greater application potential.

## Figures and Tables

**Table 1:** Performance parameters of different sensors.

Sensor Structure	Q-Factor	Sensitivity of Refractive Index (nm/RIU)	FOM (RIU^−1^)	Reference
Cylindrical silicon disk with splitting gap	54,757	746	18,650	[14]
V-shaped TiO_2_ antennas	5126	186.96	721	[52]
Photonic crystal metasurface	2000	178	445	[53]
Two semicircular cylinders’ metasurface	3210	265	883	[54]
Silicon nanoblock array metasurface	7894	171	804	[55]
Silicon disk with tilted split gap	10,135	262	2183	This work

## Data Availability

Data are contained within the article.
